# Retraction: Prognostic Role of Secretory Clusterin in Multiple Human Malignant Neoplasms: A Meta-Analysis of 26 Immunohistochemistry Studies

**DOI:** 10.1371/journal.pone.0285567

**Published:** 2023-05-04

**Authors:** The PLOS ONE Editors

After this article was published, similarities were noted between this article and submissions by other research groups which call into question the validity and provenance of the reported results. In addition, during editorial follow-up, a number of data extraction errors were found, meaning some of the conclusions are no longer supported.

In light of these issues, *PLOS ONE* cannot stand by the reliability of the reported research, and the *PLOS ONE* Editors retract this article [1].

JZ did not respond to the final editorial decision. CM, AX, KZ, ZQ, XL, CL, YH, WC, CZ, YL, SS, ZW, and BL either did not respond directly or could not be reached.
